# Screening and identification of protein interacting with goose astrovirus

**DOI:** 10.3389/fcimb.2025.1595736

**Published:** 2025-05-13

**Authors:** Lingling Qian, Yuwei Liu, Xiaochun Wang, Shixing Yang, Likai Ji, Xiaopeng Sun, Jianqiang Wang, Tongling Shan, Wen Zhang, Quan Shen

**Affiliations:** ^1^ Central Laboratory of Changshu Medicine Examination Institute, Changshu, Jiangsu, China; ^2^ Department of Laboratory Medicine, School of Medicine, Jiangsu University, Zhenjiang, China; ^3^ Department of Orthopaedics, Affiliated People’s Hospital of Jiangsu University, Zhenjiang, China; ^4^ Intensive Care Unit, Jintan District Hospital of Traditional Chinese Medicine, Changzhou, China; ^5^ Shanghai Veterinary Research Institute, Chinese Academy of Agricultural Sciences, Shanghai, China; ^6^ Institute of Critical Care Medicine, The Affiliated People’s Hospital, Jiangsu University, Zhenjiang, China

**Keywords:** astrovirus, VOPBA, LMH cell, HSPA5, protein molecular docking

## Abstract

**Introduction:**

Goose Astrovirus (GoAstV), a recently identified member of the Astroviridae family in China, predominantly affects goslings, resulting in substantial economic losses to the goose farming industry due to its high infection and mortality rates. Currently, the infection mechanism and pathogenesis of GoAstV remain unknown.

**Methods:**

Given this, the Viral Overlay Protein Blot Assay was utilized to identify and characterize proteins on the LMH (Leghorn Male Hepatoma) cell membrane that interact with Goose Astrovirus. The identities of the candidate proteins were determined via LC-MS mass spectrometry analysis, bioinformatics analysis, and UniProt database search. The interaction between HSPA5 and the astrovirus protein was further validated in vitro through Western blot and Coimmunoprecipitation experiments. Finally, bioinformatics tools such as SWISSMODEL, AlphaFold, and ZDOCK were employed to construct and analyze the docking complex model between the candidate protein and GoAstV protein, including their key binding residue sites.

**Results:**

We successfully identified a 70 kDa protein in the plasma membrane protein extracts of LMH cells and confirmed the identity of this candidate protein as HSPA5. Meanwhile, *in vitro* experiments further validated the interaction between HSPA5 and astrovirus proteins. Subsequently, we successfully predicted the docking complex model of HSPA5 protein with GoAstV protein. Further prediction of the binding residue sites revealed that seven residues of the GoAstV-P2 protein (THR124, ILE22, VAL24, TRP51, PRO66, GLN100, and VAL125) and twelve residues of the HSPA5 protein (ARG2, HIS3, LEU4, LEU6, ALA7, LEU8, LEU9, LEU10, LEU11, ASP411, VAL413, and LEU415) may be involved in the interaction between these two proteins.

**Discussion:**

Our research results have preliminarily elucidated the interaction mechanisms between viral proteins and receptors, facilitating exploration from multiple angles of the roles of candidate protein in the process of GoAstV infecting host cells. This provides a theoretical basis for further identification of GoAstV receptors and clarification of its infection mechanisms.

## 1 Introduction

Astroviruses (AstV) are categorized as positive-sense, single-stranded RNA viruses, exhibiting a round morphology with distinct surface protrusions observable under an electron microscope and measuring 25-30nm in diameter (Wu et al., 2020). In 1975, Appleton (Appleton and Higgins, 1975) et al. initially isolated AstV from the fecal samples of young children. Subsequent comprehensive research (Herrmann et al., 1991) identified this virus as a primary pathogen responsible for diarrheal symptoms in infants and newborns. The genomic structure of AstV is relatively conserved, primarily comprising 5’ and 3’ untranslated regions (UTRs), three open reading frames (ORF1a, ORF1b, and ORF2), and a polyadenylated tail (Pantin-Jackwood et al., 2011). According to the ninth report in 2014, the Astrovirus genus was further subdivided into 19 and 3 species, respectively, for mammalian and avian astroviruses.

With the advancement of diverse detection technologies, there has been a notable rise in reported cases of astrovirus infections across various species, highlighting the extensive diversity of astroviruses and their potential zoonotic risks (Bosch et al., 2014). Research indicates that AstV not only infects humans but also a broad spectrum of mammals and birds, exhibiting a propensity for genetic mutation and recombination, thereby demonstrating interspecies transmission capabilities and causing an array of diseases (De Benedictis et al., 2011; Pantin-Jackwood et al., 2011; Kang et al., 2012). Since 2017, outbreaks of a transmissible gout disease, characterized by visceral urate deposition, have sequentially emerged in major goose-producing regions of China, including Shandong, Henan, Anhui, Jiangsu, and Fujian (Zhang et al., 2017; Yin et al., 2021; Zhang et al., 2022). These outbreaks are predominantly attributed to infection with the novel Goose Astrovirus (GoAstV), which is characterized by its prolonged duration and extensive transmission range. Research suggests that GoAstV predominantly affects young geese, demonstrating high contagiousness with an incidence rate approaching 80% (Niu et al., 2018). Moreover, investigations into GoAstV infection have uncovered substantial genetic variability, resulting in the discovery of numerous new strains (Jin et al., 2018; Yang et al., 2018; Chen et al., 2020; Shen et al., 2022). Presently, astrovirus infections are widespread globally, exhibiting robust adaptability, inflicting significant economic damage on China’s goose farming industry, and posing grave risks to human health.

AstV is challenging to culture *in vitro*. Studies have demonstrated that the virus’s replication capacity is significantly enhanced following the inoculation of treated diseased material into goose embryos via the chorionic allantoic membrane for four consecutive passages (Wang et al., 2021; Zhang et al., 2022). GAstV can be isolated using LMH cells (Chen et al., 2021). However, studies have shown that no Cytopathic Effect (CPE) was observed in infected LMH cells (Zhang et al., 2018; Zhu et al., 2022). Similarly, Zhang Yujie et al. found no CPE in LMH cells after three generations of blind passages with the virus (Zhang et al., 2020). A key factor in mediating viral infection is the precise attachment of the receptor to the virus and its capability to induce viral invasion. Given the absence of commercial vaccines to prevent AstV infection, comprehending the interaction between AstV and host cell protein is essential for antiviral drug development. Presently, several methods are available for virus receptor screening. The most frequently employed is the Virus Overlay Protein Blot Assay (VOPBA). This technique entails an overnight incubation of viral fluids with cellular proteins on a PVDF membrane, leveraging the distinct binding properties of viruses to receptor proteins for preliminary receptor protein screening. Utilizing VOPBA and mass spectrometry identification techniques, researchers like Jindadamrongwech (Jindadamrongwech et al., 2004) identified HSPA5 as the receptor component for Dengue Virus Serotype 2 on HepG2 cells, further substantiating the interaction between HSPA5 and the Dengue Virus.

In this study, we utilized the VOPBA method to co-incubate isolated LMH cell membrane proteins with a concentrated solution of astrovirus, thereby successfully identifying the interaction protein HSPA5 on the LMH cell membrane in conjunction with GoAstV. We proceeded to characterize the interactions between the identified candidate proteins and the virus in greater detail. Following this, bioinformatics tools were employed to predict and analyze the molecular docking model between the candidate protein and GoAstV proteins, including their key binding residue sites. This methodology enables a comprehensive understanding of the potential role of the candidate protein as either a primary or secondary receptor in the process of GoAstV infecting host cells, offering substantial theoretical support for further identification of GoAstV receptors and elucidating the infection mechanism of GoAstV.

## 2 Materials and methods

### 2.1 Viral strains, cell strains, proteins and antibodies

The GoAstV strain (GenBank accession number: OM569656) was isolated, identified, and preserved in the liquid nitrogen tank of our laboratory. The LMH cell strain was passaged and maintained in our laboratory using DMEM F-12 supplemented with 15% FBS (Gibco) at 37°C and 5% CO_2_. The GoAstV polyclonal antibody was prepared and stored in a -80°C refrigerator in our laboratory. The HSPA5 antibody was purchased from Wuhan Sanying Biotechnology Co., Ltd. Horseradish peroxidase (HRP)-conjugated goat anti-rabbit IgG was purchased from ABclonal. Ten-day-old goose eggs were obtained from Changzhou Siji Poultry Co., Ltd. The hypersensitive developing solution was purchased from Shanghai Sangon Biological Engineering Co., Ltd.

### 2.2 Virus cultivation and concentration

In order to generate a highly virulent virus suspension, we re-inoculated virus fluids that had been previously isolated and stored in our laboratory into goose embryos. Following filtration of the viral supernatant through a 0.22µm bacterial filter, we utilized a syringe to inoculate ten-day-old goose embryos via the chorioallantoic cavity route, amounting to a total of 10 eggs. Of these, eight were designated as the positive control group, with each embryo receiving an inoculation of 200 µL of virus fluid. The remaining two were assigned to the negative control group, where each embryo was inoculated with 200 µL of sterile PBS using the same method. Subsequently, the inoculated goose embryos were incubated at a consistent temperature of 37°C and monitored twice daily over a period of seven consecutive days. In the event of embryo mortality, the deceased embryo was to be immediately removed and refrigerated, followed by the collection of allantoic fluid after a 24-hour interval. We designed primers that target a highly conserved region of the GoAstV ORF2 (GoAstV-F: TCGGACCCAATGGAAATGCT; GoAstV-R: TGTCATCAGCACTGGAACGA), which amplifies a fragment measuring 474 bp. Utilizing the allantoic fluid collected from the goose embryos as templates, we performed PCR amplification under the following conditions: initial denaturation at 95°C for 5 minutes, followed by 30 cycles consisting of denaturation at 95°C for 30 seconds, annealing at 59°C for 30 seconds, extension at 72°C for 50 seconds, a final extension at 72°C for 10 minutes, and a final hold at 16°C for 2 minutes. This process aimed to detect and select samples of allantoic fluid that exhibited strong positivity for the virus, which would then be used in subsequent experiments.

### 2.3 Extraction of LMH cell membrane proteins

Utilizing a cell scraper, LMH cells from three 10cm culture dishes, each containing approximately 10^7^ cells, were washed with PBS, collected, and centrifuged to obtain the cell deposit. Subsequently, the cell deposit was resuspended in 1mL of Reagent A (Membrane and Cytosol Protein Extraction Kit, 20127ES50), which contained PMSF, and incubated on ice for a duration of 15 minutes. The cells underwent repeated freeze-thaw cycles until the lysis efficiency achieved or surpassed 70%. In order to eliminate nuclei and unbroken cells, the lysate underwent centrifugation at 4°C and 700g for a period of 10 minutes. Following this, the supernatant was harvested and subjected to centrifugation at 14000g for a duration of 30 minutes, aiming to sediment the cell membrane fragments. Subsequent to a brief centrifugation of the resulting supernatant at 14000g for a period of 10 seconds, 200µL of Reagent B was incorporated. Thereafter, the deposit was vigorously resuspended utilizing a high-speed vortex for a duration of 5 seconds, followed by incubation on ice for a period ranging from 5 to 10 minutes. Subsequent to an additional centrifugation at the identical speed for a duration of 5 minutes, the supernatant, which now encompassed the cell membrane protein solution, was harvested and preserved at -80°C for prospective applications.

### 2.4 Screening of interacting proteins by VOPBA technique and mass spectrometry analysis

Sodium dodecyl sulfate polyacrylamide gel electrophoresis (SDS-PAGE) was conducted on various centrifugal fractions of LMH cells, including whole cell protein, cytoplasmic protein, and membrane protein. Using a semi-dry transfer technique, the bands were transferred from the gel onto a polyvinylidene fluoride (PVDF) membrane at 25V for a duration of 30 minutes. Subsequently, the membrane was blocked using 5% milk diluted in Tris-buffered saline with Tween-20 (TBST) at 37°C for a period of two hours. Afterward, the membrane was incubated overnight at 4°C with viral protein diluted in 1% milk, followed by three washes with TBST. The membrane was subsequently incubated overnight at 4°C with GoAstV polyclonal antibody (1:3000, diluted in 5% milk), washed three times, and incubated for two hours with horseradish peroxidase (HRP)-conjugated goat anti-rabbit IgG (1:5000). Following three additional washes with TBST, the membrane was developed using a hypersensitive developing solution and exposed for observation. A control group was established, and the gel was stained with Coomassie Brilliant Blue to determine the relative molecular weight of the interacting proteins on the PVDF membrane. The corresponding bands on the SDS-PAGE gel were carefully cut out and placed into pre-prepared small EP tubes, labeled, and sent to Beijing Zhongke Xinshengming Biotechnology Co., Ltd. for mass spectrometry analysis.

### 2.5 Data searching

The search engine, Proteome Discoverer 2.5, was employed to retrieve the raw data from mass spectrometry tests within pertinent databases, thereby confirming the final outcomes of the identified proteins. Utilizing the peptide sequence spectrum derived from mass spectrometry analysis, along with details regarding peptide segments and protein identification, DAVID and Venny 2.1.0 were applied to conduct a KEGG analysis of the proteins. The online analytical tools, STRING 12.0 and the UniProt database, were employed for the data analysis of proteins, facilitating the screening and identification of potential protein candidates.

### 2.6 Identification of the HSPA5 by Western blot

The HSPA5 protein, derived from LMH cell membranes, underwent separation via 10% SDS-PAGE gel electrophoresis before being transferred onto a PVDF membrane. The PVDF membrane was subsequently blocked using a 5% milk solution, which was diluted in 1×TBST, and this process was conducted at room temperature over a duration of two hours. Following three washes of the PVDF membrane, it underwent an overnight incubation at 4°C with commercially available HSPA5 rabbit PcAb, diluted at 1:500. Subsequent to another wash, the PVDF membrane was subjected to a one-hour incubation at room temperature with HRP-conjugated goat anti-rabbit IgG, which was diluted at 1:3000. Post-washing, the membrane underwent development utilizing a hypersensitive developing solution, followed by exposure for observation purposes.

### 2.7 Confirmation the interaction between HSPA5 and GoAstV by Co-immunoprecipitation experiment

In the co-immunoprecipitation experiment, 400μL of LMH cell membrane protein was incubated with 300μL of GoAstV viral fluid, diluted in PBS, at 4°C under gentle rotation for 10 hours. This was followed by the addition of the prepared rabbit PcAb and an overnight incubation at 4°C on a vertical mixer. Following this, the mixture was added to pre-treated resin (20μL rProtein A/G Agarose Resin) and incubated at 4°C with continuous rotation for an additional 4 hours. The resin was then collected via centrifugation at 8000 rpm for 3 minutes and subsequently washed three times with binding buffer. Finally, 5× SDS PAGE loading buffer was added and the mixture was boiled for 10 minutes to dissociate the protein complexes adsorbed onto the resin. The samples were then subjected to 12% SDS-PAGE gel electrophoresis, followed by Western blot analysis. A commercial HSPA5 rabbit PcAb (1:1000) was used as the primary antibody. Control rabbit IgG (1:200) served as the negative control group, while HRP-conjugated goat anti-rabbit (1:3000) was used as the secondary antibody to detect the presence of GoAstV protein. Concurrently, the mixture of membrane proteins and GoAstV proteins was designated as input.

### 2.8 Prediction of the docking model and binding residue sites between candidate proteins and GoAstV-P2 protein

In order to establish a docking model between the HSPA5 protein and the GoAstV-P2 protein, the three-dimensional structure of GoAstV-P2 was initially predicted online utilizing SWISS-MODEL (https://swissmodel.expasy.org), which was based on the protein’s amino acid sequence. Following this, the three-dimensional structure of the HSPA5 protein was predicted using AlphaFold structure prediction through Uniprot (https://alphafold.ebi.ac.uk/entry/Q90593). The molecular docking model between the GoAstV-P2 protein and the HSPA5 protein was then predicted using ZDOCK (Pierce et al., 2014), and the docking results were analyzed using the online tool PDBePISA (https://www.ebi.ac.uk/msd-srv/prot_int/). PyMOL software was used for model visualization, and the binding residues of the simulated docking model were also predicted.

## 3 Results

### 3.1 Results of PCR validation after virus amplification

**Figure 1:**
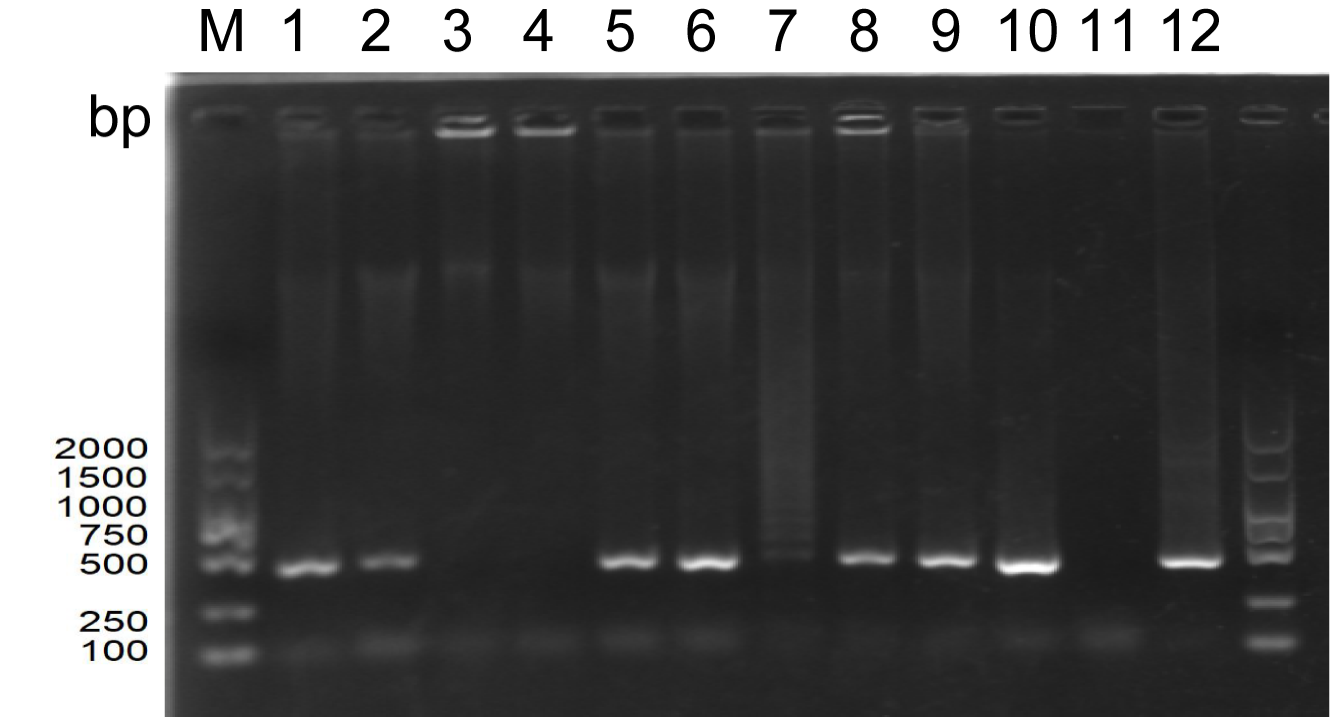
PCR electrophoretic map of the amplified allantoic luminal fluid of goose embryos (M: DNA standard molecule, bp; wells 1-2: original viral fluid retained in the laboratory; wells 3-4: PBS blank control; wells 5-12: each goose embryo inoculated with 200 µL of original viral fluid).

Our laboratory had previously isolated and stored a virus fluid, which we then re-inoculated into goose embryos. The results of the PCR electrophoresis are displayed in **Figure 1**. Upon comparing the band intensity of the original viral fluid added to wells 1 and 2, it was observed that the PCR bands from the allantoic fluid collected post-re-inoculation were relatively brighter. This suggests a further amplification and concentration of the virus. Concurrently, wells 3 and 4 served as blank controls, where an equivalent volume of PBS was inoculated into goose embryos. The absence of bands negated the potential for false positives. The diminished bands in wells 7 and 11 could potentially be ascribed to suboptimal growth conditions of the respective goose embryos.

### 3.2 Screening of interacting proteins using VOPBA technology

**Figure 2:**
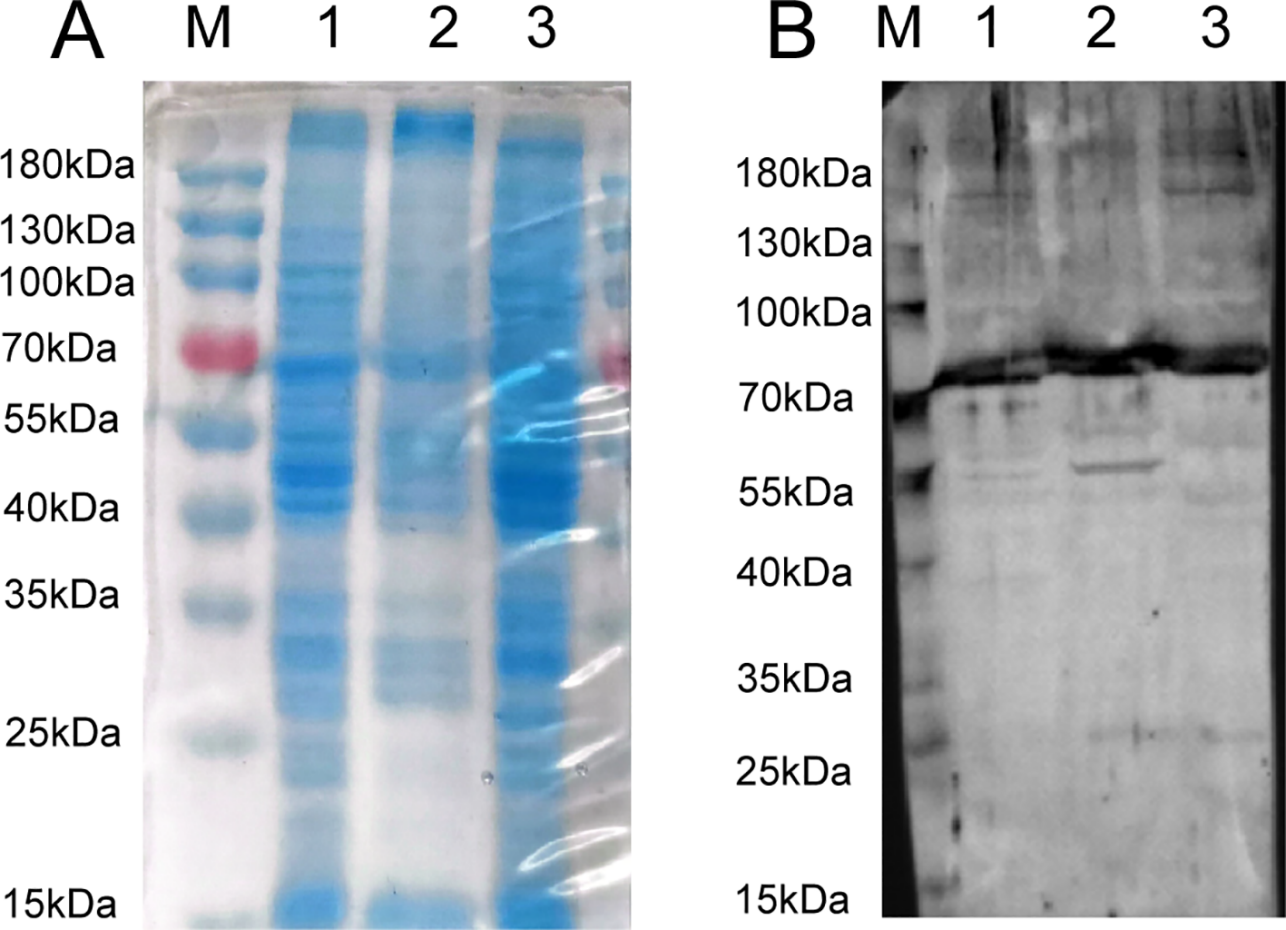
**(A)** SDS-PAGE analysis of extracted LMH cell fractions proteins; **(B)** VOPBA detection of GoAstV-interacting protein locations (about 70 kDa protein) (M: protein marker, kDa; 1: whole protein of LMH cell; 2: membrane protein of LMH cell; 3: plasma protein of LMH cell).

**Figure 2A** presents the SDS-PAGE analysis results of proteins extracted from various components of LMH cells. The figure illustrates distinct protein bands of assorted sizes in the lanes representing whole cell proteins, cell membrane proteins, and cytoplasmic proteins, which can be used for subsequent experiments. In the VOPBA assay, GoAstV intact virions were employed. A PVDF membrane, previously transferred with proteins from multiple LMH cell constituents, underwent an initial overnight incubation with the viral suspension. This process aimed to encourage specific interactions between the viral proteins and the corresponding proteins on the membrane. Following this, the membrane was treated with a prepared rabbit polyclonal antibody to identify these interacting proteins. **Figure 2B** reveals that a protein band, roughly 70kDa in molecular weight, exhibited binding affinity towards the GoAstV protein across all aforementioned protein categories. We meticulously dissected the gel region associated with the 70kDa protein derived from the cell membrane for further mass spectrometry analysis.

### 3.3 Bioinformatics analysis and mass spectrometry data analysis of host cell proteins

**Figure 3:**
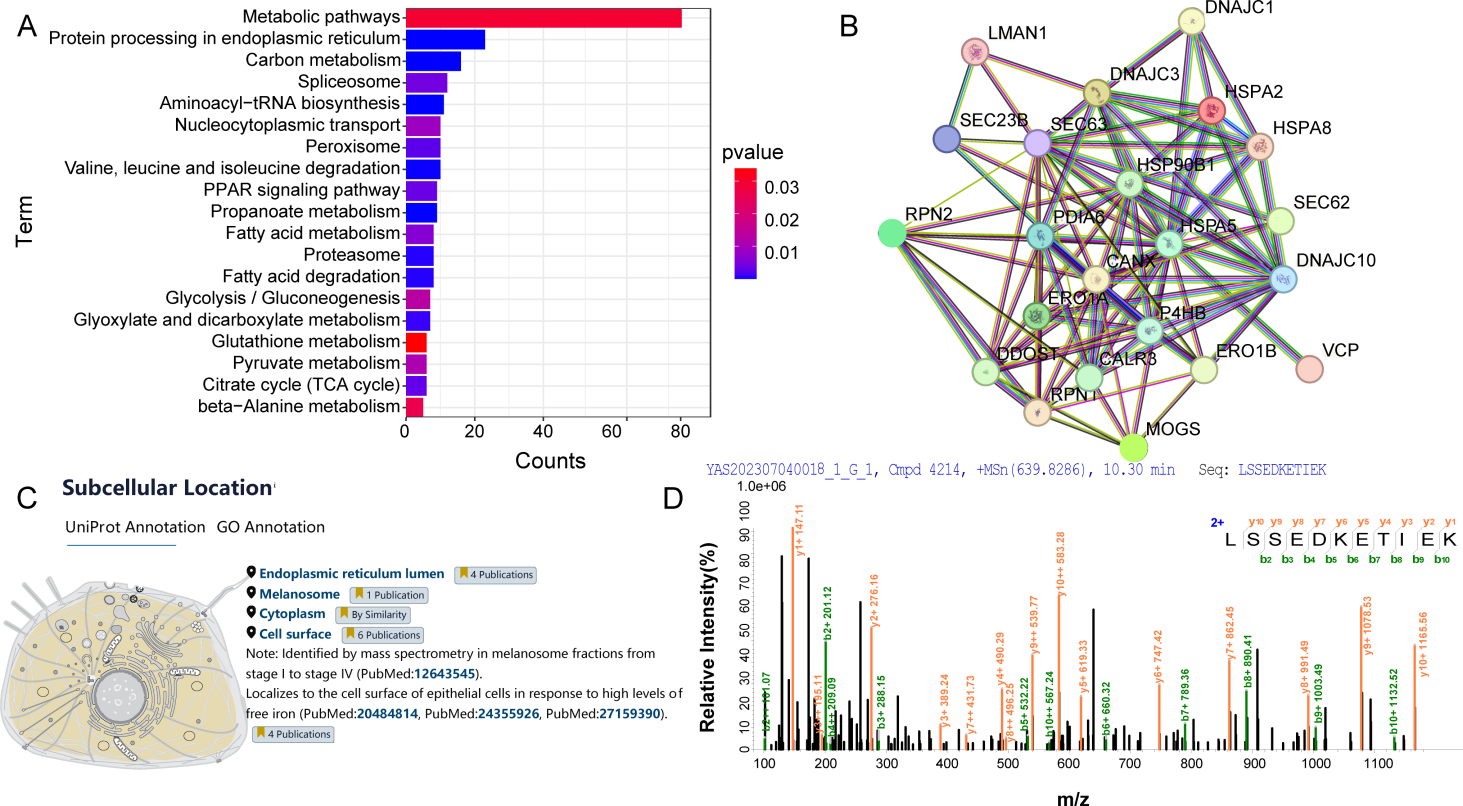
**(A)** KEGG analysis of host cell proteins interacting with GoAstV; **(B)** Interaction map of host cell proteins interacting with GoAstV; **(C)** Subcellular localization of the HSPA5 protein; **(D)** Secondary mass spectra of the candidate protein HSPA5.

Using mass spectrometry data for peptide and protein identification, gene and protein functional annotations were extracted through a search of the DAVID bioinformatics database. Concurrently, the Venny 2.1.0 online tool was used to analyze differential gene expressions and identify co-expressed genes. A subsequent KEGG enrichment analysis of cellular proteins provided insights into metabolic pathways, signaling pathways, and cellular processes. With a P-Value < 0.05, **Figure 3A** illustrates that 19 pathways were enriched, primarily involving metabolic and protein processing pathways in the endoplasmic reticulum. Utilizing the STRING 12.0 analysis tool, a medium confidence score of 0.400 was set based on Anser cygnoides species origin. This score was used to analyze potential interactions between 23 cellular protein genes and GoAstV within the protein processing pathway in the endoplasmic reticulum. As illustrated in **Figure 3B**, these proteins form a complex network of interactions, with the thickness of the edges indicating the strength of the relationships between them. After retrieving data from the UniProt database, **Figure 3C** demonstrates the subcellular localization of HSPA5 protein, primarily found in the endoplasmic reticulum and plasma membrane. The endoplasmic reticulum serves as a primary site for intracellular protein processing and modification. As a component of this organelle, HSPA5’s interactions with other proteins are essential for maintaining its functions and understanding protein synthesis and folding processes. The secondary mass spectrometry map identifies the peptides resulting from the enzymatic digestion of HSPA5 protein, characterized by fragment ions at specific mass-to-charge ratios (m/z), which create distinct peak patterns. **Figure 3D** displays the secondary mass spectrum used to identify the target band at 70kDa, composed of HSPA5 components. This helps confirm the identity of HSPA5 and rule out potential contamination or misidentification.

### 3.4 Western blot identification of candidate protein

**Figure 4:**
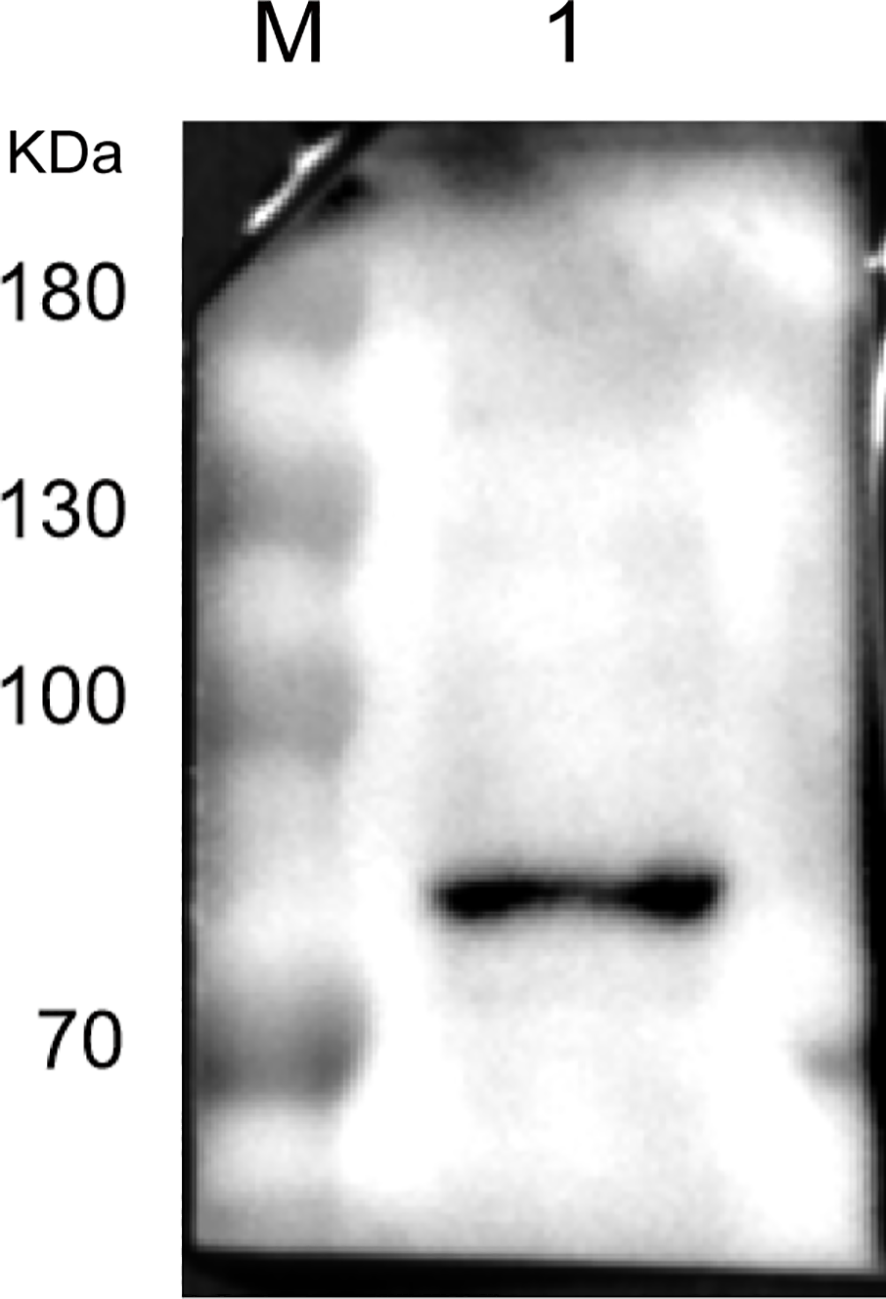
Result of Western blot identification of HSPA5 protein (M: protein marker, kDa; 1: HSPA5 protein protein).

Utilizing a commercial HSPA5 antibody as the primary antibody and an HRP-conjugated goat anti-rabbit IgG antibody as the secondary antibody, we examined the presence of HSPA5 protein in LMH cell membrane protein. As depicted in **Figure 4**, the results demonstrate a band at approximately 70kDa (note that the actual molecular weight of the HSPA5 protein is about 72kDa), thereby confirming the presence of HSPA5 protein in LMH cell membrane protein.

### 3.5 Co-IP validation of GoAstV and HSPA5 protein interactions

**Figure 5:**
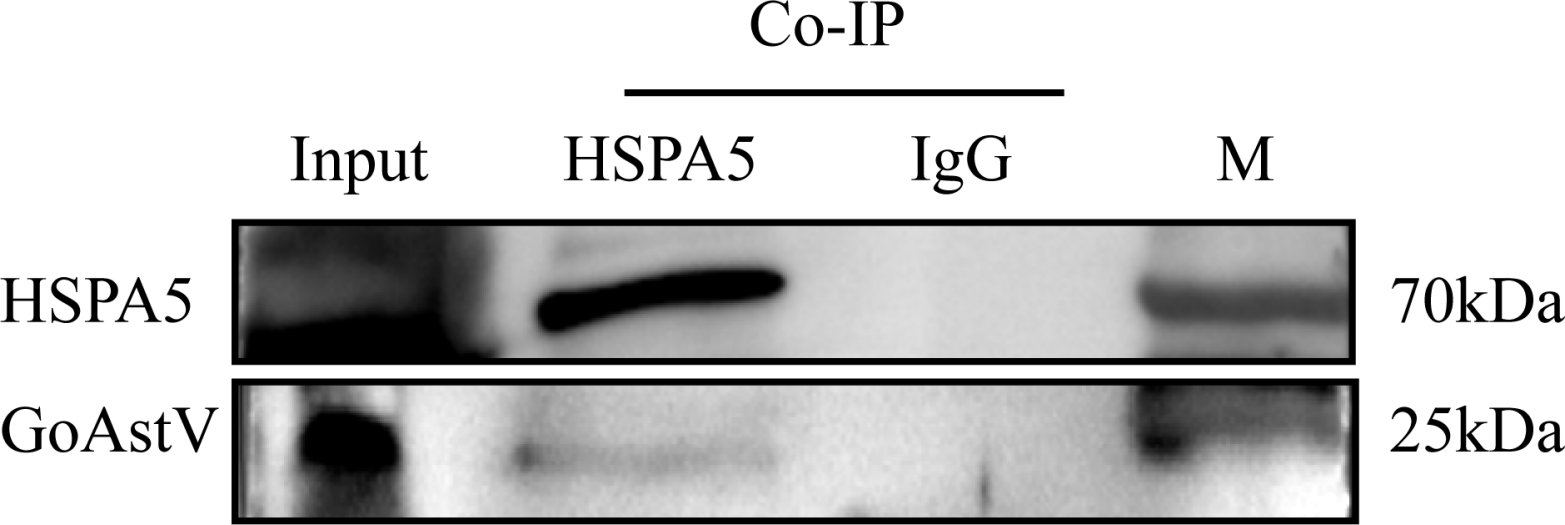
Result of immunoprecipitation assay to validate the interaction of HSPA5 with GoAstV protein (Input: Virus and membrane protein incubation solution; HSPA5: Co-IP experimental group; IgG: Co-IP negative control group).

Co-immunoprecipitation (Co-IP) assays were performed to confirm the interaction between GoAstV and HSPA5. LMH cell membrane proteins underwent overnight incubation with the viral fluid, which was subsequently subjected to immunoprecipitation utilizing the pre-prepared rabbit polyclonal antibody. The interaction was then confirmed using a commercially available HSPA5 antibody. As depicted in **Figure 5**, both the Co-IP experimental group and the input group displayed bands approximately at 70kDa and 25kDa. In contrast, the negative control group did not exhibit any bands, implying an interaction between the HSPA5 protein and the GoAstV protein.

### 3.6 3D structural model prediction of GoAstV-P2 protein and HSPA5 protein

**Figure 6:**
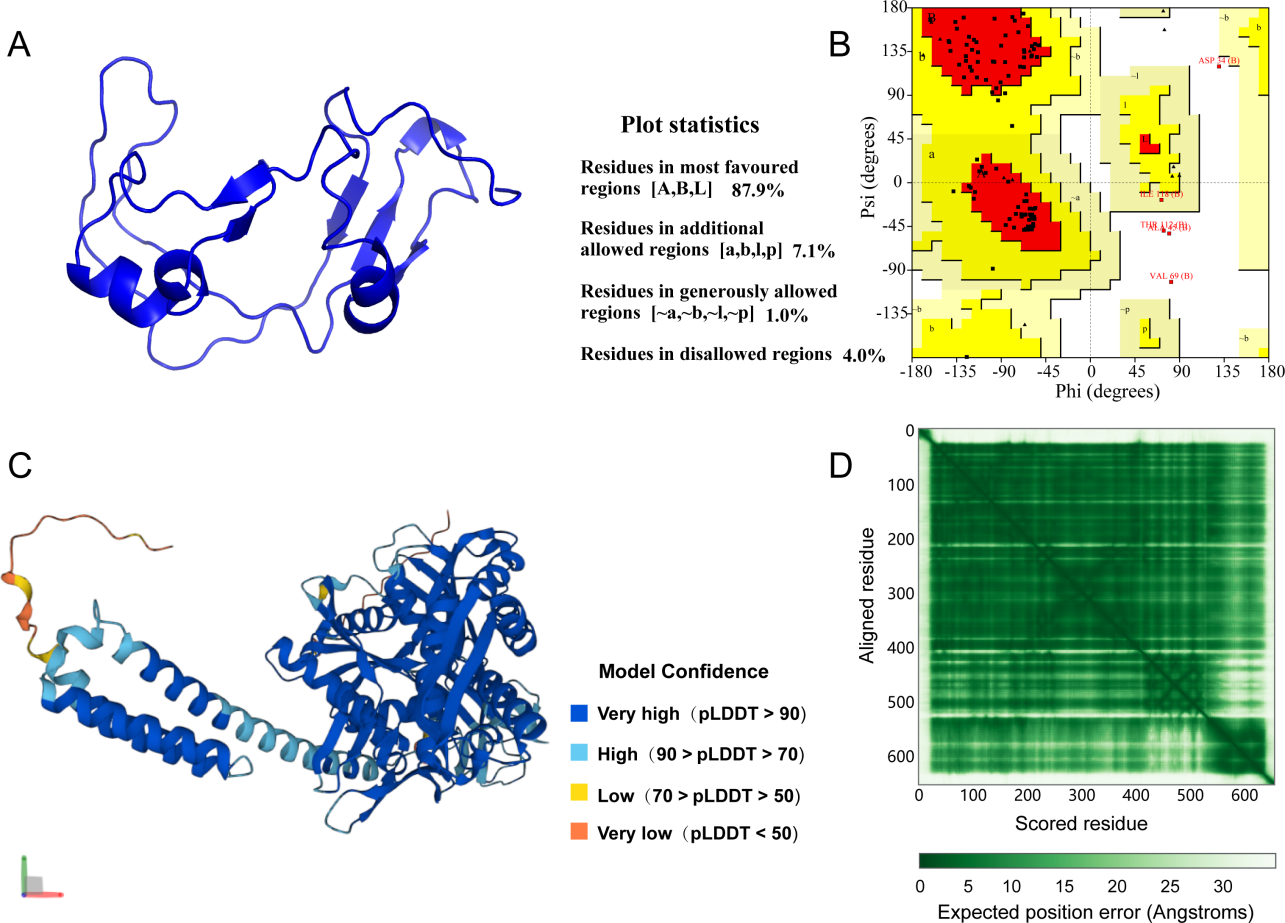
**(A)** Predicted 3D structure of GoAstV-P2 protein; **(B)** GoAstV-P2 protein ramachandran plot; **(C)** Predicted 3D structure of HSPA5 protein; **(D)** HSPA5 protein prediction alignment error (PAE) plot.

The homology modeling of the GoAstV-P2 protein was performed using SWISS-MODEL, and its predicted 3D structure was shown in **Figure 6A**. The stereochemical quality of the protein structure model was assessed by analyzing the distribution of mainchain dihedral angles φ and ψ, as presented in the Ramachandran plot in **Figure 6B**. Over 96% of the residues fell within the most favorable and allowed regions, indicating a high degree of confidence in the model. The per-residue model confidence score (pLDDT) generated by AlphaFold generally ranges from 0 to 100, with regions below 50 pLDDT possibly representing isolated unstructured areas. **Figure 6C** presented the predicted 3D structure of the HSPA5 protein, showing that the majority of residues have a pLDDT > 90, suggesting a high model confidence score. The PAE plot displayed in **Figure 6D** further demonstrates that the protein 3D structure prediction is both effective and accurate.

### 3.7 Bioinformatics prediction of the interaction between GoAstV-P2 protein and HSPA5 protein

**Figure 7:**
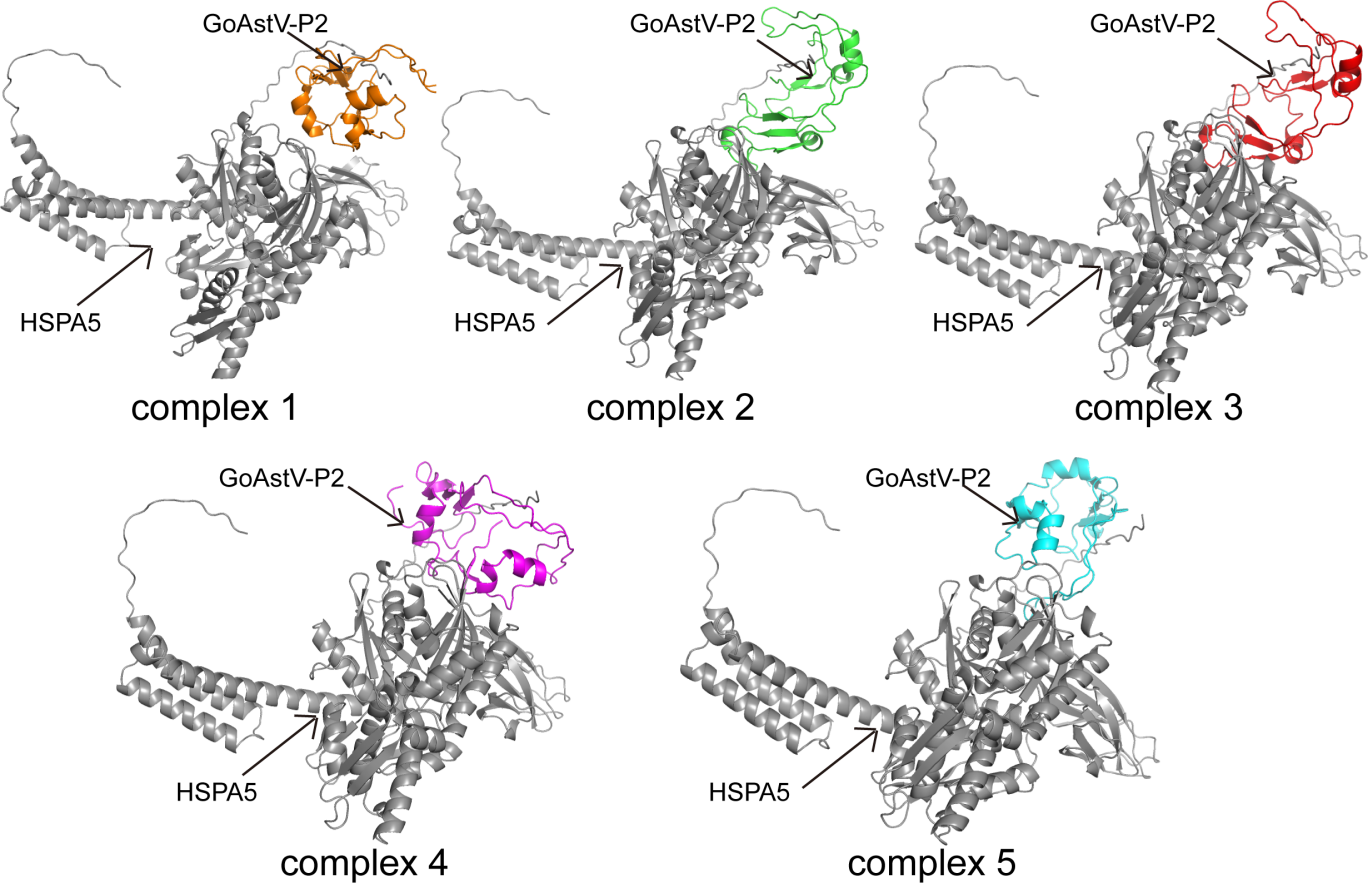
Models of molecular docking of GoAstV-P2 protein with HSPA5 protein ranked in the top five by ZDOCK score (Gray represents HSPA5 protein; orange, green, red, purple, and cyan all represent GoAstV-P2 protein).

The docking models of GoAstV-P2 and HSPA5 protein molecules were generated using the ZDOCK rapid Fourier transform algorithm. The resulting docking models were then ranked according to their ZDOCK scores, where higher scores corresponded to increased model reliability. **Figure 7** displayed the top five scoring docking models, revealing diverse binding modes between the two protein molecules. The best docking complex model was analyzed using the online tool PDBePISA. The Interface List (**Supplementary Table S2**) provides information on the interface area size (in Å²) of the protein interaction surface, as well as the free energy (ΔiG in kcal/mol) under this docking mode. A lower free energy value of -11.9 indicates that the docking model is structurally stable. The Interface Summary (**Supplementary Table S3**) lists the total number of atoms and residues involved in the docking, with structure1 having 1133 atoms and 119 residues, and structure2 having 5067 atoms and 652 residues, respectively. The Hydrogen bonds table (**Supplementary Table S4**) and Salt bridges table (**Supplementary Table S5**) provide detailed information about non-covalent interactions such as hydrogen bonds and salt bridges in the best docking complex model. By analyzing these data, we can conclude that the complex has structural stability and specific amino acid residues interact with each other through hydrogen bonds and salt bridges to maintain its function.

**Figure 8:**
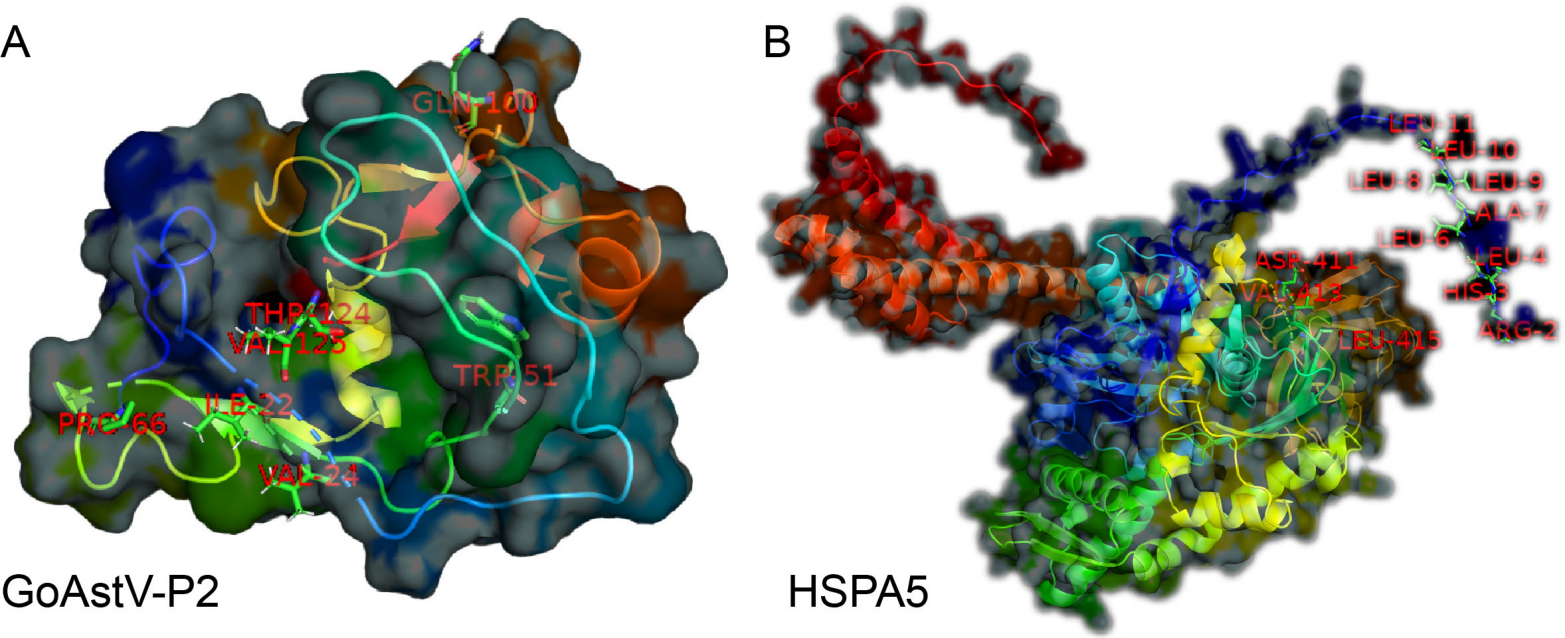
Prediction of the functional residues involved in the binding between GoAstV-P2 and HSPA5 (**A**: Seven residues from the GoAstV-P2 protein: THR124, ILE22, VAL24, TRP51, PRO66, GLN100, and VAL125; **B**: twelve residues from the HSPA5 protein: ARG2, HIS3, LEU4, LEU6, ALA7, LEU8, LEU9, LEU10, LEU11, ASP411, VAL413, and LEU415)

Additionally, the Interfacing residues tables (**Supplementary Tables S6**, **S7**) list all the residues involved in the interaction from both proteins, the types of chemical bonds they participate in (e.g., hydrogen bonds H, salt bonds S, disulfide bonds D, covalent bonds C), and the ratio of the buried surface area of the residues in the interaction interface. More vertical lines indicate a higher proportion of buried surface area. Based on this, further prediction of binding residue sites revealed that seven residues from the GoAstV-P2 protein (THR124, ILE22, VAL24, TRP51, PRO66, GLN100, and VAL125) and twelve residues from the HSPA5 protein (ARG2, HIS3, LEU4, LEU6, ALA7, LEU8, LEU9, LEU10, LEU11, ASP411, VAL413, and LEU415) are likely involved in the interaction between the two proteins (**Figure 8**).

## 4 Discussion

Astrovirus (AstV) is recognized as a principal pathogen responsible for diarrheal symptoms in infants and newborns. It is distinguished by its significant genetic variability and propensity for zoonotic diseases, which can result in a variety of illnesses (Herrmann et al., 1991; Bosch et al., 2014). Currently, the infection mechanism of AstV, particularly regarding the infectious receptors, is still not understood. Viruses rely on suitable host cells for infection and replication, which begins with the specific binding between viral surface ligands and host cell surface molecules or receptors. Within the cell, the interaction between viral and host intracellular proteins is essential for completing the viral replication cycle. To enhance the prevention of AstV infections and identify potential therapeutic targets, it is crucial to deepen our understanding of the molecular mechanisms underlying the interactions between GoAstV and its host cells. The GoAstV-P2 protein, which serves as both a major antigenic determinant and a receptor-binding protein, could potentially regulate host cell mechanisms to foster an environment favorable for viral replication (Méndez et al., 2002; Krishna, 2005; Dryden et al., 2012). Consequently, the screening and identification of host cell membrane surface and intracellular proteins that interact with the virus are crucial for understanding the detailed mechanisms of viral infection, replication, and transmission.

Current research on virus-host protein interactions primarily employs two approaches: protein and genetic methods. The main technical means include VOPBA technology, co-immunoprecipitation, yeast two-hybrid technology, phage display technology, affinity chromatography, GST pull-down technology, and gene chip technology, to name a few (Bourgonje et al., 2020). VOPBA represents a conventional and extensively utilized technique for viral receptor screening, which is founded on the Western blot methodology. Under defined *in vitro* conditions, this technique facilitates the identification and isolation of previously unidentified receptor proteins via the specific binding of viral proteins to these receptors (Navaratnarajah et al., 2009). This approach has proven successful in the receptor screening for pathogens such as the dengue virus, measles virus, and rabies virus (Maisner et al., 1994; Karger and Mettenleiter, 1996; Salas-Benito and del Angel, 1997). After obtaining concentrated viral fluid, to ensure the purity of membrane proteins and minimize cell membrane damage induced by conventional mechanical disruption, this study used a cell membrane protein extraction kit to extract LMH cell membrane proteins. Subsequently, during the fundamental research phase focusing on goose astrovirus cellular membrane receptors, VOPBA technology enabled the successful screening of a candidate protein, exhibiting a relative molecular mass of 70kDa, on the LMH cell membrane. This protein demonstrated specific binding with GoAstV particles. Through LC-MS/MS mass spectrometry, the protein band was conclusively identified as glucose-regulated protein 78 (GRP78) (Deshmukh et al., 2010).

GRP78, also known as HSPA5 protein, is a heat shock protein widely present in the endoplasmic reticulum and cytoplasmic membrane. As a primary chaperone protein, it participates in various cellular processes including the cellular stress response, protein folding and degradation, regulation of Ca^2+^ homeostasis, and acting as a sensor for endoplasmic reticulum stress (Li and Lee, 2006; Ni and Lee, 2007; Liu et al., 2010). Recent studies have highlighted the multiple functions of the protein, linking it to cancer, inflammatory diseases, and autoimmune disorders (Quinones et al., 2008; Gonzalez-Gronow et al., 2009; Ni et al., 2011). Although the primary function of HSPA5 is as a chaperone protein, numerous studies have shown that a majority of HSPA5 proteins are manifested on the cell surface (Delpino and Castelli, 2002; Shin et al., 2003; Jindadamrongwech et al., 2004). Indeed, there have been documented interactions between the HSPA5 protein and diverse viral proteins, such as the dengue virus protein (Jindadamrongwech et al., 2004), the Japanese encephalitis virus envelope protein E (Nain et al., 2017), and the Zika virus envelope protein E (Khongwichit et al., 2021). To the best of our knowledge, this constitutes the inaugural report detailing an interaction between the HSPA5 protein and the GoAstV-P2 protein. Such investigations imply that HSPA5 could potentially act as a somewhat conserved viral receptor or co-receptor, facilitating the adsorption and subsequent penetration of various viruses into cells. Within the spectrum of identified cell membrane proteins, a preliminary selection of HSPA5 protein for further receptor validation tests was made based on comprehensive analysis of protein scores, peptide matching degrees, and binding protein positional data within the cell. A Western blot assay substantiated the existence of HSPA5 among LMH cell membrane proteins, and a Co-IP experiment affirmed the interaction between the GoAstV protein and the HSPA5 protein.

As bioinformatics and computational biology rapidly advance, predicting protein 3D structure and its application in protein-protein molecular docking has become crucial methods for studying protein functions. SWISS-MODEL, a widely used automated homology modeling tool (Waterhouse et al., 2018), constructs the 3D model of target proteins based on the sequences of homologous proteins with known structures. It features advanced template recognition technology and precise model construction algorithms. Model accuracy is typically evaluated using Ramachandran plots (Laskowski et al., 2012), where a reasonable model should have the majority of residues falling within the most favorable and allowed regions. In this study, SWISS-MODEL was utilized to predict the 3D structure of the GoAstV-P2 protein. The Ramachandran plot result indicated that over 96% of the residues in the model were located in the most favorable and allowed regions (Sobolev et al., 2020), suggesting a high degree of model reliability. Moreover, studies suggest that protein sequences from the AlphaFold database, when clustered using the MMseqs2 algorithm (Steinegger and Söding, 2018), are viable for predicting protein tertiary structures. Utilizing AlphaFold, we predicted the tertiary structure of the HSPA5 protein based on its amino acid sequence. During the prediction process, AlphaFold first performs feature extraction on the sequence through a CNN, subsequently capturing long-range dependencies using a Transformer. Following this, a sequence of structural refinement and energy minimization procedures are executed to ascertain the forecasted tertiary structure of the HSPA5 protein, demonstrating notable model reliability. These findings align with those of Jumper et al., who demonstrated AlphaFold’s capability to accurately forecast protein structures in most instances, encompassing both the protein’s main chain and side chains (Jumper et al., 2021). One output of the AlphaFold system is the Predicted Aligned Error (PAE), which can be used to evaluate the reliability of the relative positions and orientations of different parts within a protein’s 3D model (Varadi et al., 2021). In the PAE plot, dark green blocks indicate good predictions (low error); conversely, light green blocks suggest poorer predictions (high error) (Jumper et al., 2021). In our study, the predominance of dark green blocks in the PAE plot signified diminished model error, thereby suggesting enhanced reliability and precision.

Protein-protein molecular docking is a critical technique that simulates the relative spatial positioning and orientation of two or more protein molecules. The objective is to identify the most energetically optimized and stable complex structure. This process reveals the nature and magnitude of intermolecular forces, thereby establishing a theoretical basis for subsequent functional analysis. Following the prediction of the three-dimensional structural models for the GoAstV-P2 and HSPA5 proteins, the docking model between these two proteins was predicted using ZDOCK. The ZDOCK score (Mintseris et al., 2007), also known as the Z-score, measures the agreement between the model and the native structure. A higher ZDOCK score indicates more reliable docking results. In this study, we present the top five docking models, as ranked by their ZDOCK scores. These models illustrate the intricate nature of protein interactions via diverse binding modes. The analysis of the best docking complex model using the online tool PDBePISA indicates that the predicted docking model has high stability. Utilizing PyMOL (Wu et al., 2022) enabled the visualization of the docking interface between GoAstV-P2 and HSPA5 proteins, allowing predictions of specific residues likely implicated in the binding mechanism, thereby enriching our comprehension of protein interaction mechanisms.

## 5 Conclusion

In brief, this study screened and verified the protein HSPA5 on LMH cell membranes as a potential interactor with the GoAstV protein. Through bioinformatic approaches, we predicted the binding mode and key binding residues between HSPA5 and the GoAstV-P2 protein, laying the theoretical foundation for further investigation into the role of HSPA5 as an interaction protein or co-receptor in GoAstV infection mechanisms.

## Data Availability

The original contributions presented in the study are included in the article/**Supplementary Material**. Further inquiries can be directed to the corresponding authors.

## Supplementary material

Supplementary Table 1 Mass Spectrometry Identification Information Sheet.

Supplementary Table 2 Detailed information of the interfaces list in the best docking complex model.

Supplementary Table 3 Detailed information of the interface summary in the best docking complex model.

Supplementary Table 4 Detailed information of the hydrogen bonds in the best docking complex model.

Supplementary Table 5 Detailed information of the salt bridges in the best docking complex model.

Supplementary Table 6 Detailed information of the interfacing residues in Structure 1 of the best docking complex model.

Supplementary Table 7 Detailed information of the interfacing residues in Structure 2 of the best docking complex model.
